# Investigation of the Effects of Three-Dimensional Modeling Techniques on Degenerative Rotoscoliosis Surgery

**DOI:** 10.7759/cureus.13075

**Published:** 2021-02-02

**Authors:** Ismail Kaya, Ilker Deniz Cingöz, Meryem Cansu Şahin, Emirhan Bozoğlan

**Affiliations:** 1 Neurosurgery, Kutahya Health Sciences University, Kutahya, TUR; 2 Medical Physics, Kutahya Health Sciences University, Kutahya, TUR; 3 Bioengineering, Kutahya Health Sciences University, Kutahya, TUR

**Keywords:** rotoscoliosis, 3d printing, pedicle screw

## Abstract

Objectives

The present study aimed to compare patients in whom an operation plan was prepared before surgery using the three-dimensional (3D) modeling technology with the application of freehand screws using magnetic resonance imaging (MRI) and computed tomography (CT) scan images.

Methods

The printings and modelings were established in the Training and Research Center. Of 40 patients, 20 underwent surgery with 3D printing (Group 1) and 20 with the freehand technique (Group 2). The surgeries were performed by the same surgeons. Moreover, 5-mm pedicle screws were located in 122 vertebrae in 20 patients in whom 3D modeling was used and in 124 vertebrae in 20 patients in whom this modeling technique was not used.

Results

The mean time of screw insertion was 2.9 ± 1.2 minutes in the experimental group and 4.7 ± 2.3 minutes in the control group. While the mean amount of bleeding was 7.4 ± 4.1 ml in the experimental group, it was found to be 39.6 ± 14.2 ml in the control group. When the locations of the screws in the experimental group were evaluated, it was seen that 106 (86.9%) screws were ‘excellent’ and 16 (13.1%) screws were ‘good.’ When the placement of 124 pedicle screws in the control group was evaluated, it was found that 100 (80.6%) screws were ‘excellent,’ 20 (17.8%) screws were ‘good,’ and two (1.6%) screws were ‘poor.’

Conclusion

The use of the improved 3D technology in the neurosurgery field is advantageous for surgeons, as it decreases the preoperative preparation phase, length of operation, and risk of complications.

## Introduction

Lumbar degenerative scoliosis is the curvature of the lumbar spine after it completes its development [1]. Curvatures of more than 10° on the anteroposterior radiographs of the spine of adult patients are called "adult lumbar scoliosis." The prevalence of adult lumbar scoliosis varies between 8.3% and 68% [2]. Its incidence increases with advancing age. Although it is generally seen in women, it is seen around the age of 70 years [3]. A pedicle screw is one of the most important tools in lumbar degenerative scoliosis treatment [4]. However, the abnormal vertebral anatomy (abnormal pedicle anatomy) in these patients causes difficulties in pedicle screw applications [5]. Various techniques have been introduced to ensure the safe and accurate placement of transpedicular screws [6]. Pedicular screw malposition rates varying between 21.1% and 39.8% have been reported in transpedicular screw applications with a conventional technique [7]. Because the pedicle is adjacent to the spinal canal and surgery is conducted in a narrow anatomic area, the accuracy and safety of using transpedicular screws are essential to prevent complications. Surgeons can make preoperative plans with direct radiography, computed tomography, and spine models produced by three-dimensional (3D) printers for patients who are planned to undergo spinal surgery [8]. With the support of three-dimensional printers, radiological images were transformed into concrete objects, and it was observed that the duration of surgery was shortened with preoperative planning [9]. With the shortening of the duration of the surgery, blood loss and the need for blood transfusion have also been reduced [9]. At the same time, shortening the duration of the surgery decreased the use of fluoroscopy during surgery and reduced the radiation dose to which both the patient and the surgeon were exposed. Models produced with 3D printers supported by radiological imaging allow both doctors and patients to understand the existing pathology and to make a preoperative plan [8]. In this study, in the surgical treatment of lumbar degenerative rotoscoliosis, it was aimed to compare the cases in which the surgery plan was prepared using the 3D modeling technology before surgery and the free-hand technique applied by making plans only from the patient magnetic resonance imaging (MRI) and computed tomography (CT) images.

## Materials and methods

Ethics committee approval

Ethics committee approval was received for this study from the Ethics Commission ("2020-04/09" no decree dated 08.07.2020).

Patient selection

This study was conducted with CT images of 40 randomized patients who were operated on with the diagnosis of lumbar degenerative rotoscoliosis in the neurosurgery clinic between July 2018 and August 2019. Of 40 patients, 20 underwent surgery with 3D printing (Group 1) and 20 with the freehand technique (Group 2). The surgeries were performed by the same surgeons. This study had the following inclusion criteria: 1) age between 50 and 65 years, 2) Cobb angle 30 degrees and above or curvatures progressing more than 10 degrees in their follow-up, 3) patients with listhesis of more than 6 mm or curvature of more than 3 mm in their follow-up. Patients who had previously undergone spine surgery, preoperative radiation, chemotherapy, or had recurrent tumors were excluded from the study.

Digital design and 3D printing

**Figure 1:**
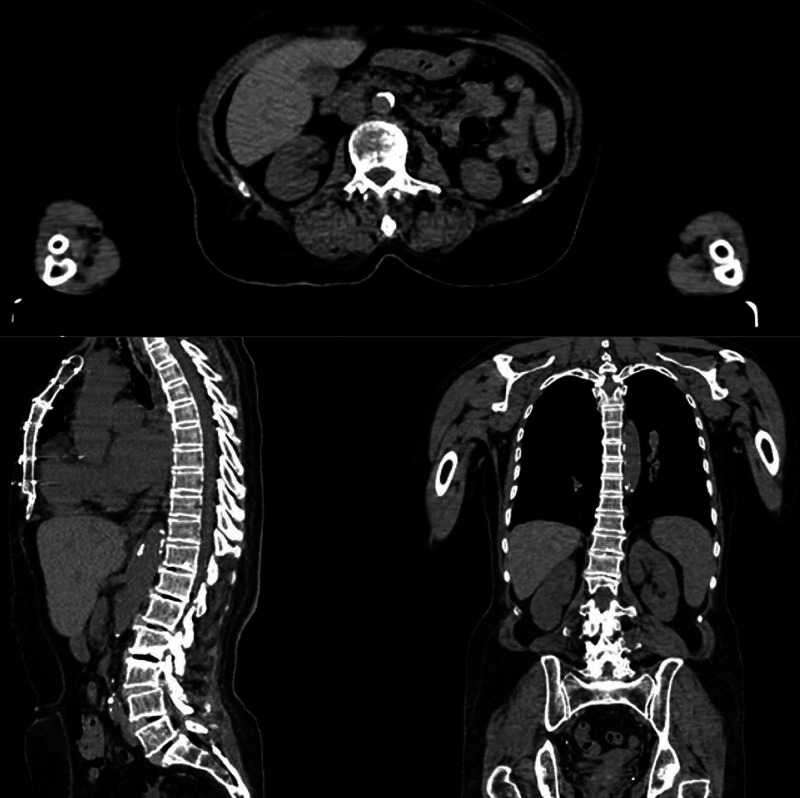
CT images for three-dimensional modeling of a patient with rotoscoliosis CT: computed tomography

Preoperative computed tomography (CT) images of 20 patients diagnosed with lumbar degenerative rotoscoliosis in the neurosurgery clinic were used for 3D models. Preoperative digital imaging and communications in medicine (DICOM) images of each patient were reconstructed using 32-channel CT at a slice thickness of 0.625 mm and a planar resolution of 0.35 mm (Aquilion™ Large Bore CT, Canon Medical Systems, Tustin) (Figure 1).

**Figure 2:**
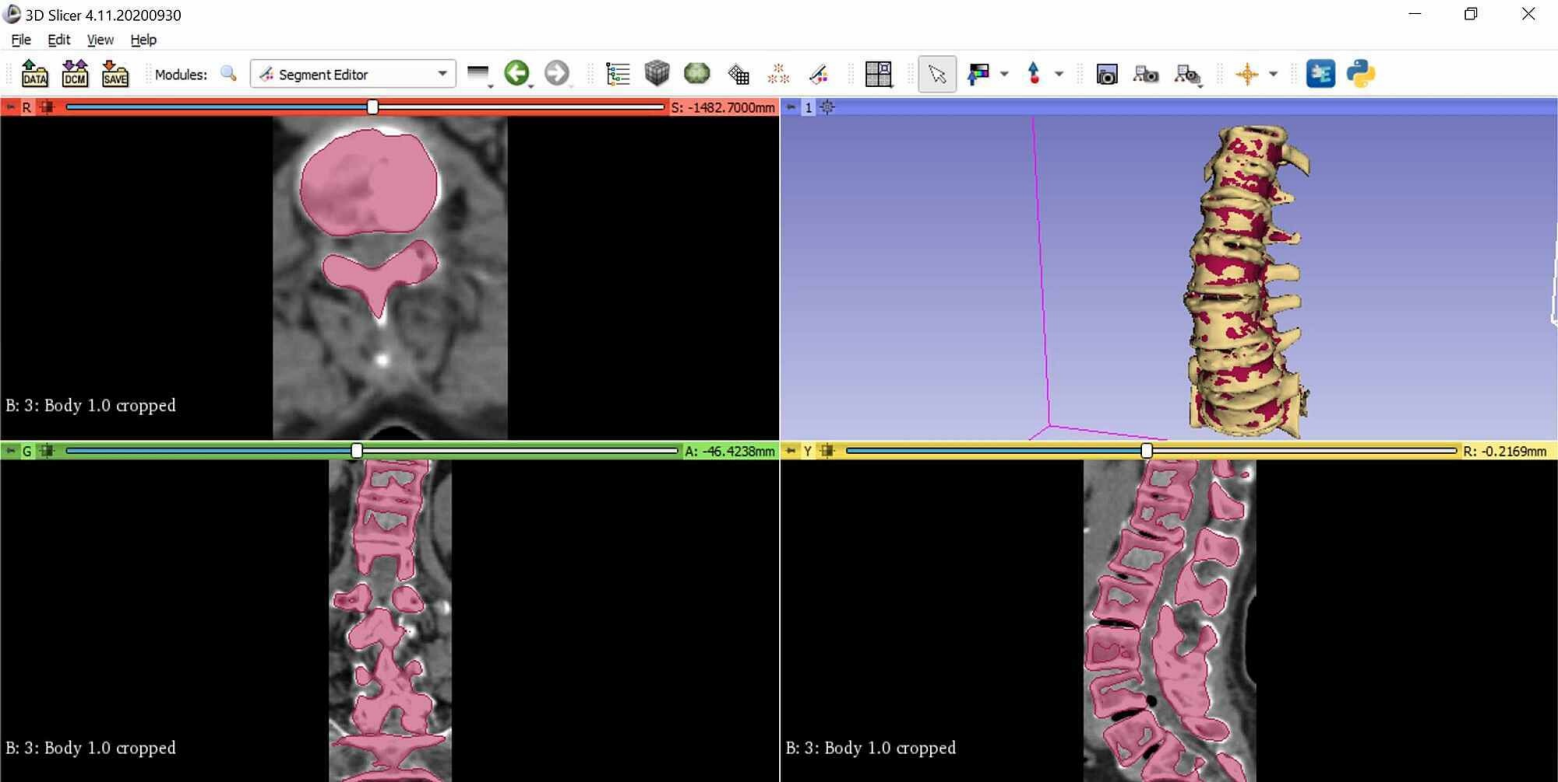
3D modeling of vertebrae using the 3DSlicer program 3D: three dimensional

CT images containing approximately 450 sections for each model were transferred to the 3DSlicer (version 4.10.1, Boston) program to create a 3D vertebral model. Using this software, the images were used to create 3D models of the vertebral region related to the complex surface treatment method (Figure 2). 

**Figure 3:**
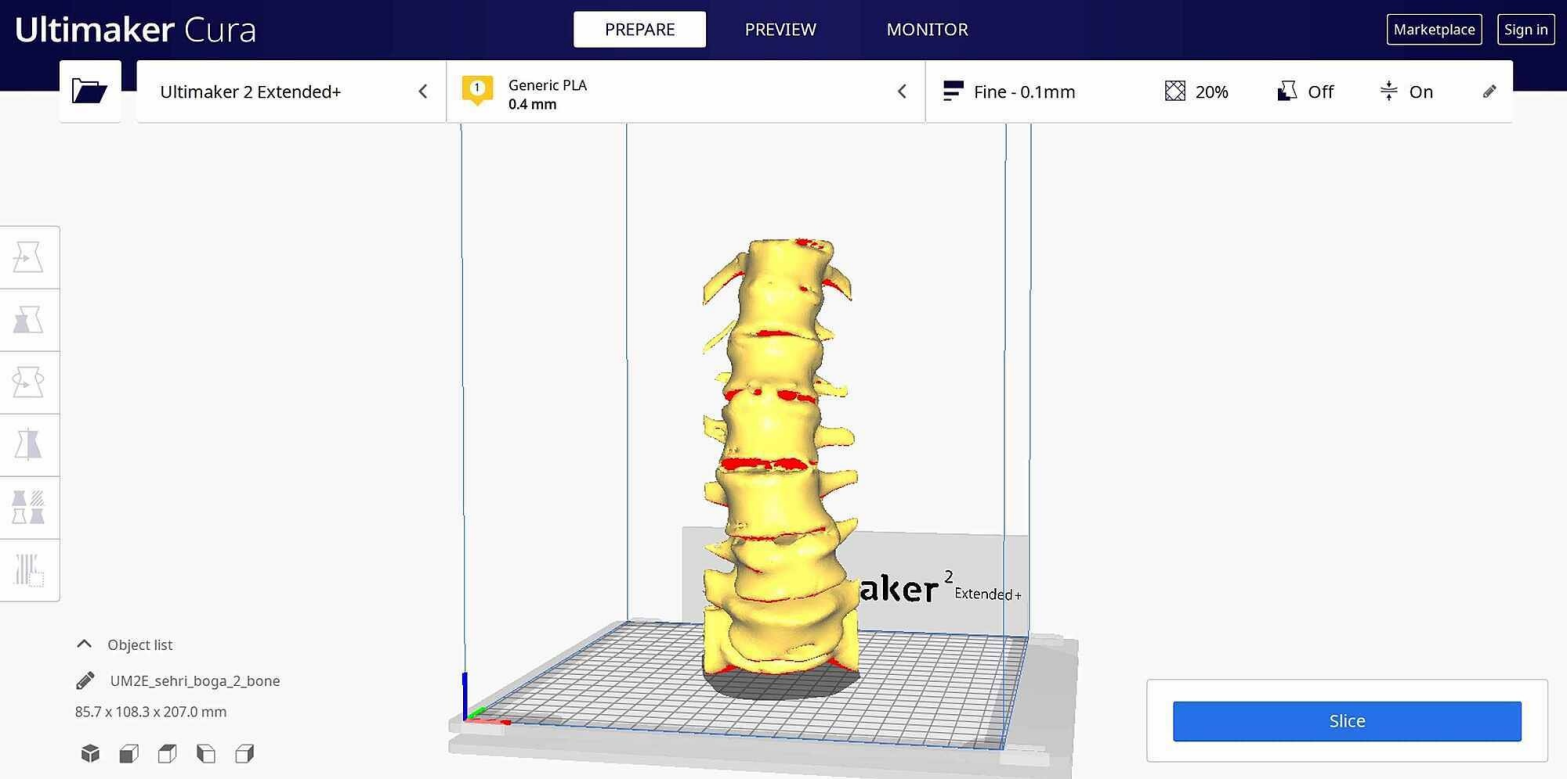
Slicing the model using the Ultimaker Cura program prior to 3D printing

3D model data were saved in stereolithography (STL) format and transferred to Ultimaker Cura (version 4.7.1) (Ultimaker B.V., Utrecht, Netherlands) software. The following printing parameters were used for Ultimaker 2 Extended 3D printer and polylactic acid (PLA) in Ultimaker Cura software for the printing of preoperative models: 0.4 mm nozzle diameter, 200 °C nozzle temperature, 70 °C build plate temperature, and 70% filling rate (Figure 3). Preoperative planning studies were carried out by the relevant surgeon on vertebra models created using preoperative DICOM images.

Operational methods

**Figure 4:**
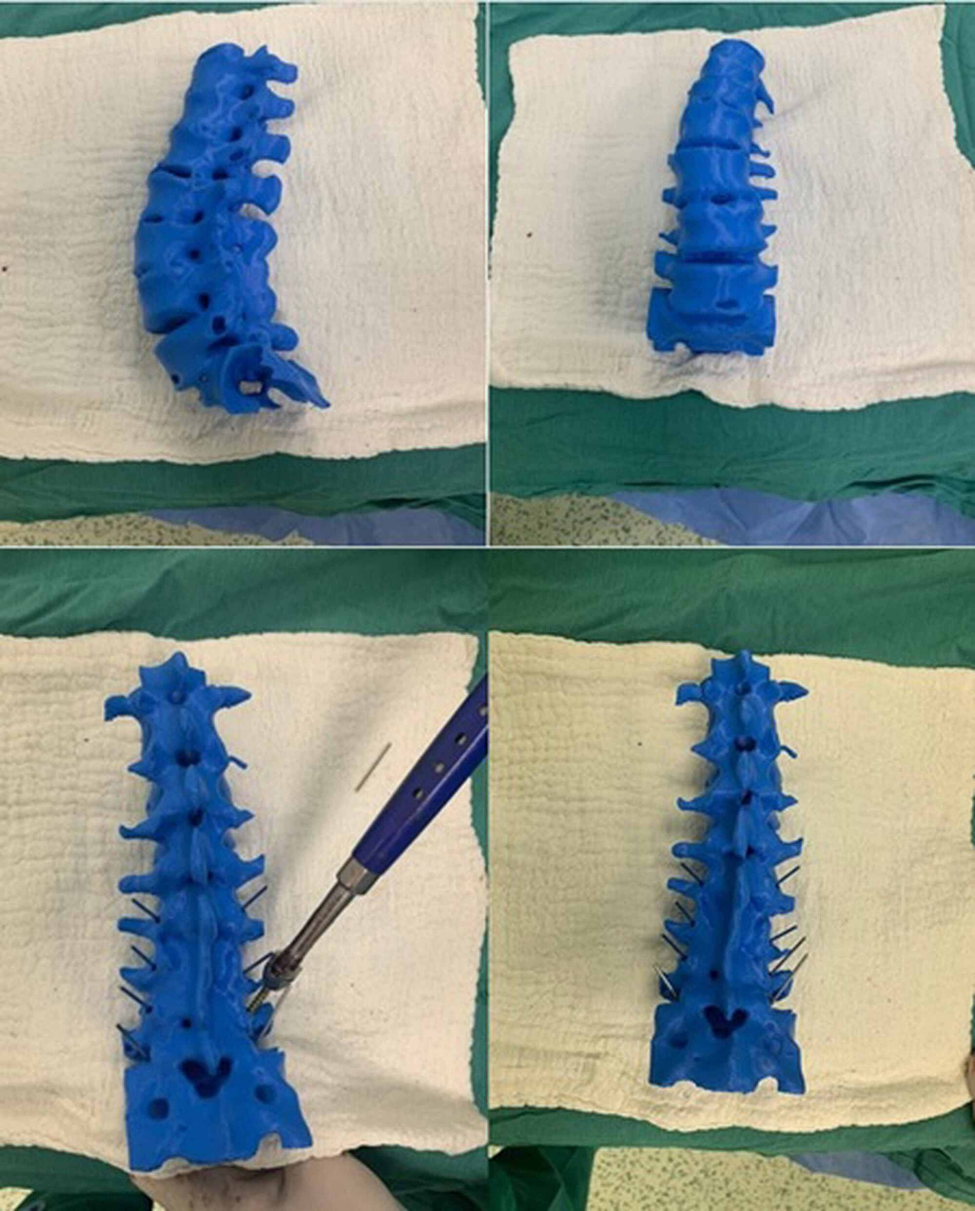
Preop vertebral simulations and pedicle screw placement

**Figure 5:**
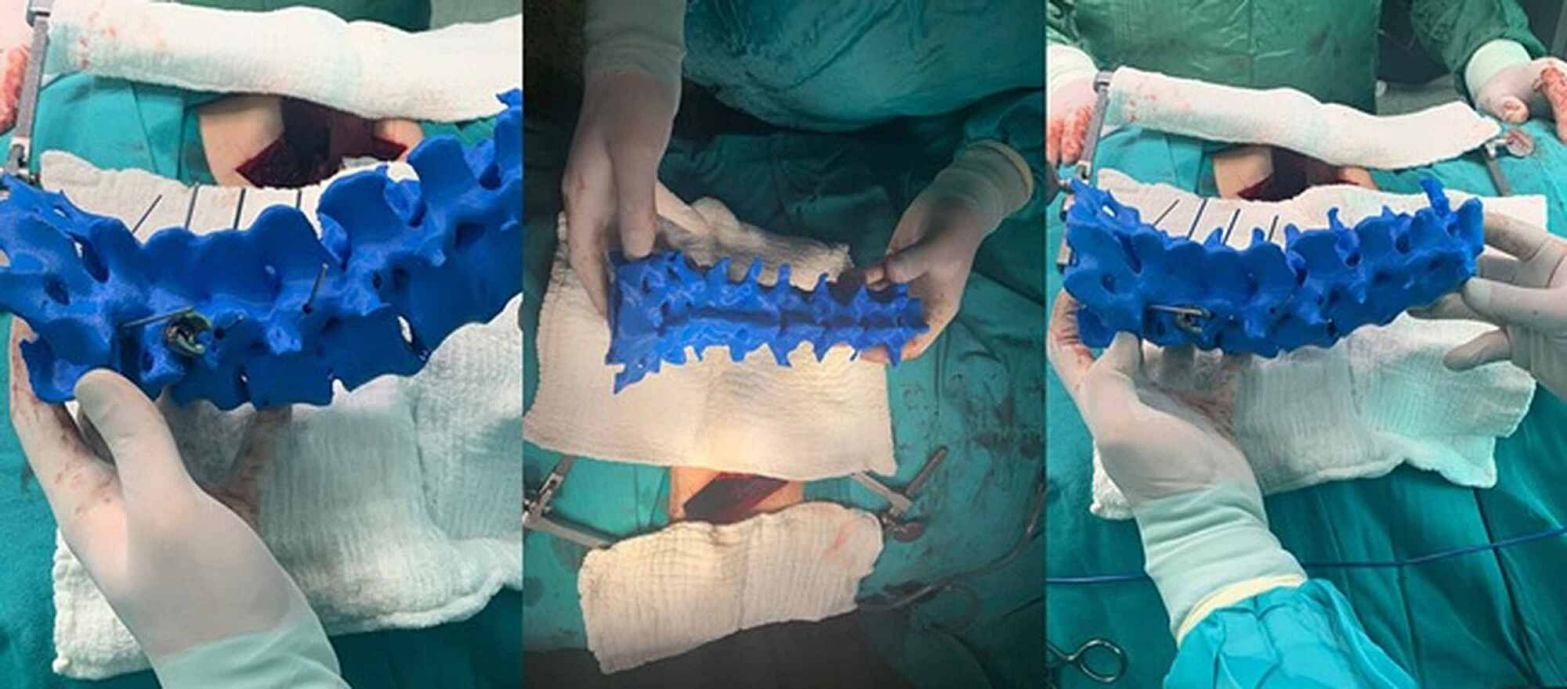
Rotoscoliosis operation performed with the 3D model

The patients to be operated on with the help of the 3D printing and modeling method were planned with preoperative patient-specific models (Figure 4). Patient-specific full-scale spine models of these patients at the time of surgery were available for reference (Figure 5). Pedicle screws were placed from anatomic regions previously determined with the planning made and checked by fluoroscopy. In the control group patients, pedicle screws were placed using the free-hand technique and under fluoroscopic control with 2D C-arm-scopy.

Evaluation of efficacy

Intraoperative bleeding amount, pedicle screw insertion time, and correct screw placement data in both groups were recorded. Intraoperative bleeding was calculated by subtracting the volume of fluid used for flushing from the total fluid volume in the suction bag. The time of insertion of each pedicle screw was recorded. A control CT scan was performed after the operation. Screw malpositions and violations of the medial and lateral walls of the pedicles were noted. The correct placement of the screws was evaluated using the Richter method [10-11]. According to this method, screw placement is evaluated in three categories: 1) Excellent: the screws were completely positioned in the pedicle; 2) Good: only screw thread went through the cortex of the pedicle isthmus of the vertebral arch (less than 1/4 of the screw diameter), without danger to surrounding nerves and vessels; 3) Poor: the screws clearly penetrated the bone cortex of the vertebral arch's pedicle isthmus (more than 1/4 of its diameter), which may damage surrounding nerves and vessels [11].

Statistical analysis

The statistical analysis was performed using the Statistical Package for the Social Sciences (SPSS) 24.0 (IBM Corp.; Armonk, NY) software. Data are presented as mean ±SD (x±s), and intergroup comparison was done with the independent-samples t-test. The enumeration data were expressed as a ratio, and intergroup comparison was done with the chi-square test; p=0.05 was used as the statistics inspection standard.

## Results

It was observed that both groups were similar in terms of gender and age distribution. All the patients were followed up for three months. No obvious complications of nerves, vessels, or viscera were reported in either group. The location of the screws was evaluated by performing a postoperative lumbar CT scan. A total of 122 pedicle screws were placed in the experimental group and 124 in the control group. The average fluoroscopy numbers were 48.45 ± 12.23 in patients in whom 3D modeling was used and 84.55 ± 24.42 in patients in whom the screws were applied using the freehand technique (Table 1).

**Table 1:** Comparative demographic and surgical data of both groups

	Group 1: Non-3D Printing	Group 2: 3D Printing
N		
Total screws placed	124	122
Gender, n (%)		
Female	12 (%60)	12 (%50)
Male	8 (%40)	8 (%50)
Number of fluoroscopic images	84.55±24.42	48.45 ± 12.23
Screw insertion time (min)	2.9 ± 1.2	4.7 ± 2.3
Surgical blood loss (ml)	740±98	396±68

The mean time of screw insertion was 2.9 ± 1.2 minutes in the experimental group and 4.7 ± 2.3 minutes in the control group, and this difference was found to be statistically significant (p<0.05). While the mean amount of bleeding was 740±98 ml in the experimental group, it was found to be 396±68 ml in the control group, and this difference was found to be statistically significant (p<0.05).

A total of 122 pedicle screws were placed in patients operated on with the help of 3D modeling. When the locations of the screws in the experimental group were evaluated, it was seen that 106 (86.9%) screws were ‘excellent’ and 16 (13.1%) screws were ‘good.’ When the placement of 124 pedicle screws in the control group was evaluated, it was found that 100 (80.6%) screws were ‘excellent,’ 20 (17.8%) screws were ‘good,’ and two (1.6%) screws were ‘poor.’ There was no statistically significant difference between the two groups between excellent and good screw placement rates (p> 0.05) (Table 2).

**Table 2:** Screw placement accuracy (it was calculated using the Richter method)

Screw Placement Category	Non-3D Printing (n=124 screws)	3D Printing (n=122 screws)
Excellent	100 (80.6%)	106 (86.,9%)
Good	22 (17.7%)	16 (13.,1%)
Poor	2 (1.7%)	-

A significant difference was found between the cases performed using 3D modeling and the cases not used, in terms of the use of fluoroscopy (p<0.001). While the mean number of fluoroscopy was 48.45 ± 12.23 in patients operated with three-dimensional modeling, it was found that the mean number of fluoroscopy was 84.55 ± 24.42 in cases with freehand screw application.

## Discussion

Lumbar degenerative scoliosis is the curvature of the lumbar spine after completing its development [1]. Curvatures of more than 10° on the anteroposterior radiographs of the spine of adult patients are called "adult lumbar scoliosis." The prevalence of adult lumbar scoliosis varies between 8.3%-68% [2]. Although the use of instruments is common in the surgical treatment of degenerative rotascoliosis, this technique is accompanied by prolonged operation times, increased blood loss during surgery, and increased risk of neurological complications. Spine models produced with 3D printers using computer-aided modeling via CT provide the surgeon with preoperative simulation.

In our study, simulations were performed by making special modeling for each patient before surgery using the 3D printing method, and these patients were compared with the patients who were applied screws to the vertebrae with a freehand application without simulation using only preoperative MRI and CT images.

Qiang et al. operated on nine patients with ankylosing spondylitis and kyphoscoliosis using a personalized 3D printing guide. Sixty-five point nine (65.9) degrees of improvement in the osteotomy site was detected in nine patients, who were followed up for an average of 21.4 months. There were no patients with misplaced pedicle screws or neurological complications [12]. Perez-Mananes et al. applied 3D modeling in the orthopedic clinic and compared the duration of surgery, duration of fluoroscopy, and deformity correction rates with this method. As a result, they showed that preoperative modeling shortened the duration of the surgery and decreased the margin of error [13]. In the study of Karlin et al., which included 17 pediatric patients, personalized 3D printer models were used to correct spinal deformity in children. In the group using pre-op 3D printer models, surgical time, use of fluoroscopy, and blood loss were found to be less [14]. In the study of Garg et al., 10 patients were operated on with the help of 3D printing (Group 1) and the other 10 patients were operated on with the free-hand technique (Group 2). The medial violation was found statistically significant in the free-hand group (p = .005). A total of 57 fluoroscopy shots (5.7 per patient) were obtained in the 3D printing group while a total of 119 fluoroscopy shots were found to be significantly higher in the free-hand group [15]. Fei Wang et al. reported in their retrospective study that 3D modeling before surgery reduces the amount of anesthesia, operation time, and the number of fluoroscopies performed during the case [16]. In our study, a statistically significant difference was found between the patients who used the 3D modeling technique and the other patients in terms of operation time and blood loss. It has been observed that surgery times are shorter and blood loss during the operation is less in patients who underwent surgery with 3D modeling. It was found that the use of fluoroscopy was significantly less in patients who underwent 3D modeling. This is an important issue for both the patient and the surgical team. Klein et al., in their article published in 2015, determined a roadmap for spine imaging. In the article, the need for a systematic approach for the correct interpretation of frequently used spine imaging methods is mentioned [17]. Hong et al. examined the effectiveness of CT in comprehending 3D deformities. In the study, direct radiography and computed tomography of the entire spine were taken before and after the operation. They measured vertebral rotation, rib hump index, and sternal shift rates on the images. They found that CT is a useful method in determining 3D deformities, planning treatment, and evaluating surgical results [18]. The study of Minyi Yang et al. included 76 patients (Group A) operated on with the traditional freehand technique and 62 patients (Group B) who were treated with pedicular fixation supported by 3D printing. There was no significant difference in clinical outcomes for any of the follow-up time points for the Japanese Orthopedic Association (JOA), visual analog scale (VAS), or neck disability index (NDI) scores between the two groups. However, compared to Group A, Group B was found to have better results for atlantoaxial pedicular fixation (P=0.003), shorter operative times (P=0.001), and less blood loss (P = 0.037). Unlike the navigation system, which is expensive for most hospitals, a 3D printing model can be used as a common tool to guide upper cervical surgery [19]. Hyun Jin Park et al. created a total of 20 3D printing models from 10 volunteer patients. On these 3D models, two surgeons with no experience of free-hand pedicle screw instrumentation were guided by an experienced surgeon. Each surgeon placed 10 pedicle screws in the 3D model for each lumbar spine. The results of the second spine model were compared with those in the first model to assess the learning effect. A total of 37/200 (18.5%) screws perforated the pedicle cortex by an average of 1.7 mm (range 1.2-3.3 mm). However, it was found that the last half of the models violated less than the previous half (10/100 vs. 27/100, p <0.001). In the spine model, 10 pedicle screw instrument time was determined as 42.8 ± 5.3 minutes in the first 10 vertebra models and 35.6 ± 2.9 minutes for the last 10 vertebrae models. Significantly less time was spent in the last 10 spine models than in the previous 10 models (p <0.001). As a result, it has been observed that the full-size 3D printed spine model is an excellent tool for pedicular screwing for beginners in the freehand technique [20]. In a prospective study by Alparslan et al., 134 pedicle screws placed with 3D model simulation in patients with adolescent idiopathic scoliosis were evaluated. Postoperatively, all screws were evaluated and classified by CT into class 1 (correct), class 2 (faulty), and class 3 (deviation). Mean medial malposition was 0.5 ± 0.8 and 0.4 ± 0.6 mm on the concave and convex sides, and the mean lateral malposition was 1.4 ± 2.3 and 0.8 ± 1.3 mm. 117 screws were accepted as class 1, 14 screws as class 2, and 3 screws as class 3, and positional accuracy was determined in 92.5% of all screws inserted. No complications related to pedicle screws were detected in the study [21].

CT-based navigation systems are used to guide the placement of pedicle screws on the spine. However, the accuracy of the systems is questioned, and the failure rate is high (8.5-11%). Its use is not common due to its disadvantages such as intraoperative position changes and spinal instability, lack of real-time navigation, and high cost [22].

With the use of 3D printing in spine surgeries, the production of guide plates, and the provision of preoperative simulation, the accuracy of operations has increased. The fact that the accuracy is not affected by the intraoperative position and the higher reliability of guide plates provides superiority to navigation systems. Providing preoperative simulation and using the model as a guide during surgery reduce the surgeon's margin of error and operation time [23].

In our study, no statistically significant difference was found between the freehand group and the operated group with 3D modeling when screw malpositions were compared. However, no screw was detected in the 'poor' category in the group operated with 3D modeling. This showed us that preoperative simulations reduce the possibility of screw malposition.

Our study allowed the application of the model before the surgical operation. In this way, the surgical operation was simulated in a kind of virtual environment with the modeling study. The models obtained have been guiding the surgeon on which vertebra and how to insert screws. Vertebral models produced with the 3D printing method provide optimal surgical planning, short operation times, and successful scoliosis surgery.

## Conclusions

In conclusion, the use of the improved 3D technology in the neurosurgery field is advantageous for surgeons, as it decreases the preoperative preparation phase, length of operation, and risk of complications.

## References

[1] Aebi M (2005). The adult scoliosis. Eur Spine J.

[2] Carter OD, Haynes SG (1987). Prevalence rates for scoliosis in US adults: results from the first National Health and Nutrition Examination Survey. Int J Epidemiol.

[3] Silva FE, Lenke LG (2010). Adult degenerative scoliosis: evaluation and management. Neurosurg Focus.

[4] Ledonio CG, Polly DW, Jr. Jr., Vitale MG, Wang Q, Richards BS (2011). Pediatric pedicle screws: comparative effectiveness and safety. A systematic literature review from the Scoliosis Research Society and the Pediatric Orthopaedic Society of North America Task Force. J Bone Joint Surg Am.

[5] Kim YJ, Lenke LG, Cheh G, Riew KD (2005). Evaluation of pedicle screw placement in the deformed spine using intraoperative plain radiographs: a comparison with computerized tomography. Spine (Phila Pa 1976).

[6] Puvanesarajah V, Liauw JA, Lo SF, Lina IA, Witham TF (2014). Techniques and accuracy of thoracolumbar pedicle screw placement. World J Orthop.

[7] Castro WH, Halm H, Jerosch J, Malms J, Steinbeck J, Blasius S (1996). Accuracy of pedicle screw placement in lumbar vertebrae. Spine (Phila Pa 1976).

[8] Li Z, Xu R, Li M, Li J, Liu Y (2015). Three-dimensional printing models improve understanding of spinal fracture—a randomized controlled study in China. Sci Rep.

[9] Yang M, Li C, Li Y (2015). Application of 3D rapid prototyping technology in posterior corrective surgery for Lenke 1 adolescent idiopathic scoliosis patients. Medicine (Baltimore).

[10] Richter M, Mattes T, Cakir B (2004). Computer-assisted posterior instrumentation of the cervical and cervico-thoracic spine. Eur Spine J.

[11] Chen H, Wu D, Yang H, Guo K (2015). Clinical use of 3D printing guide plate in posterior lumbar pedicle screw fixation. Med Sci Monit.

[12] Tu Q, Ding HW, Chen H (2019). Three-dimensional-printed individualized guiding templates for surgical correction of severe kyphoscoliosis secondary to ankylosing spondylitis: outcomes of 9 cases. World Neurosurg.

[13] Perez-Mananes R, Burro JA, Manaute JR, Rodriguez FC, Martin JV (2016). 3D surgical printing cutting guides for open-wedge high tibial osteotomy: do it yourself. J Knee Surg.

[14] Karlin L, Weinstock P, Hedequist D, Prabhu SP (2017). The surgical treatment of spinal deformity in children with myelomeningocele: the role of personalized three-dimensional printed models. J Pediatr Orthop B.

[15] Garg B, Gupta M, Singh M, Kalyanasundaram D (2019). Outcome and safety analysis of 3D-printed patient-specific pedicle screw jigs for complex spinal deformities: a comparative study. Spine J.

[16] Wang F, Li CH, Liu ZB (2019). The effectiveness and safety of 3-dimensional printed composite guide plate for atlantoaxial pedicle screw: A retrospective study. Medicine (Baltimore.

[17] Klein JP (2015). A practical approach to spine imaging. Continuum (Minneap Minn.

[18] Hong JY, Suh SW, Easwar TR, Modi HN, Yang JH, Park JH (2011). Evaluation of the three-dimensional deformities in scoliosis surgery with computed tomography: efficacy and relationship with clinical outcomes. Spine (Phila Pa 1976).

[19] Yang M, Zhang N, Shi H (2019). Three-dimensional printed model-assisted screw installation in treating posterior atlantoaxial internal fixation. Sci Rep.

[20] Park HJ, Wang C, Choi KH, Kim HN (2018). Use of a life-size three-dimensional-printed spine model for pedicle screw instrumentation training. J Orthop Surg Res.

[21] Senkoylu A, Cetinkaya M, Daldal I, Necefov E, Eren A, Samartzis D (2020). Personalized three-dimensional printing pedicle screw guide: innovation for the surgical management of patients with adolescent idiopathic scoliosis. World Neurosurg.

[22] Gelalis ID, Paschos NK, Pakos EE (2012). Accuracy of pedicle screw placement: a systematic review of prospective in vivo studies comparing free hand, fluoroscopy guidance, and navigation techniques. Eur Spine J.

[23] Chen H, Guo K, Yang H, Wu D, Yuan F (2016). Thoracic pedicle screw placement guide plate produced by three-dimensional (3-D) laser printing. Med Sci Monit.

